# Identification of candidate targets and mechanisms involved in miRNA regulation in multiple myeloma

**DOI:** 10.1186/s12957-021-02482-1

**Published:** 2022-01-26

**Authors:** Yang Yang, Rong Ding, Rui Wang

**Affiliations:** Department of Clinical Haematology, Lianshui People’s Hospital Affiliated with Kangda College of Nanjing Medical University, No. 6, Hongri East Avenue, Lianshui County, Huai’an, 223400 Jiangsu Province China

**Keywords:** Multiple myeloma, miRNAs, Candidate target, Immune response

## Abstract

**Background:**

Multiple myeloma (MM) is a complex disease affected by many factors. The recognition of miRNA networks is helpful for specific detection and personalised treatment.

**Methods:**

mRNA expression profiles were obtained from GSE39754 and GSE87830, and differentially expressed mRNAs (DEmRs) between MM and controls were identified. The intersection of the two sets of DEmRs in GSE39754 and GSE87830 was identified as common mRNAs, and enrichment analysis was subsequently performed. Moreover, we analysed differentially expressed miRNAs (DEmiRs) between MM and controls in GSE87830. A regulatory network of target mRNAs related to the overall survival of MM patients was then constructed.

**Results:**

In this study, a total of 356 common mRNAs were identified that were significantly enriched in neutrophil-mediated immunity, Th17 cell differentiation and PI3K-Akt signalling pathways. Moreover, we identified 103 DEmiRs and predicted 91 differentially expressed mRNAs as target mRNAs. Cox regression analysis was used to screen 14 target mRNAs that significantly affected the survival of MM patients. In the constructed integrated regulatory network, HIF1A and THBS1 were found to participate in Th17 cell differentiation and PI3K-Akt signalling pathways.

**Conclusion:**

These findings improve the understanding of the regulatory mechanisms of MM. Genes that are part of integrated regulatory networks may represent candidate targets for MM treatment.

## Introduction

Multiple myeloma (MM) is a common malignant tumour derived from plasma cells in the bone marrow [1]. Since plasma cells develop to the final functional stage of B lymphocytes, MM can also be classified as B lymphocyte lymphoma [2]. Currently, the World Health Organisation classifies MM as a type of B cell lymphoma [3]. MM is characterised by abnormal proliferation of bone marrow plasma cells accompanied by excessive production of monoclonal immunoglobulin or light chain (M protein). A small number of patients may have MM that does not produce M protein [4]. The occurrence of MM is often accompanied by extensive osteolytic damage, hypercalcaemia, anaemia and kidney damage [5]. The production of normal immunoglobulin is inhibited, so various bacterial infections are prone to occur. According to statistics, the mortality rate of MM is 48.5% [6]. The 2019 Cancer Survey showed that amongst 32,110 patients with myeloma in the USA, 12,960 patients died, and the incidence and mortality of the disease were much higher in men than in women [7]. Many effective treatments have been developed in clinical studies over the past few decades [8]. MM easily relapses, causing MM to still be defined as an incurable disease [9]. Therefore, it is particularly important to explore new treatment methods for MM and identify specific targets for MM treatment.

MicroRNAs (miRNAs) are a class of noncoding single-stranded RNA molecules with a length of approximately 22 nucleotides. They are widely expressed in animals and plants and play important roles in both physiological and pathological processes [10]. To date, studies have revealed that changes in miRNA expression are present in a variety of tumour types [11]. Moreover, studies have shown that miRNAs have a regulatory effect on tumour progression [12]. Meanwhile, miRNAs can also represent early warning indicators of tumour occurrence, treatment and prognosis [13]. In MM research, investigators have revealed the application of miRNAs in the diagnosis of tumour development and potential tumour treatment, as well as part of the mechanism for evaluating the prognostic status of tumours. In addition, some researchers have studied the effects of miRNAs on tumours in other animal models [14]. These studies also indicated that the application and transformation of miRNAs in MM has important potential [15]. However, although researchers have revealed a large number of miRNAs associated with MM, there are still no specific miRNAs for the diagnosis, treatment or prognosis of MM. For that reason, we collected RNA sequencing data from normal and MM tissues from a public database to determine changes in differential genes. This study provides a theoretical basis for future research on miRNAs in the treatment of MM.

## Materials and methods

### Data collection

All data were obtained from the Gene Expression Omnibus (GEO) database. GSE39754 included the mRNA expression profile of CD138 purified myeloma plasma cells from 170 newly diagnosed MM patients and CD138 purified plasma cells from 6 healthy donors. GSE87830 included mRNA and miRNA expression profiles of CD138 purified myeloma plasma cells from 95 newly diagnosed MM patients and 4 noncancer samples.

### Differential analysis

The limma R package [16] was utilised to identify differentially expressed mRNAs (DEmRs) and differentially expressed miRNAs (DEmiRs) between MM and controls. For these analyses, |log2-fold change (FC) | >1 and *P* < 0.05 were considered screening conditions.

### Functional and pathway enrichment

For enrichment of mRNAs, the clusterProfiler R package [17] was used. These included Gene Ontology (GO) and Kyoto Encyclopedia of Genes and Genomes (KEGG) pathways. Biological process (BP), cellular component (CC) and molecular function (MF) were included in GO terms. Enrichment results with *P* < 0.05 were used as the threshold of statistical significance.

### Target prediction and Cox regression

The miRTarget database was used to predict the target mRNAs regulated by the identified miRNAs. Cox regression analysis for genes in the MMRF-CoMMpass database was performed to identify mRNAs that significantly influenced MM patient prognosis.

## Results

**Fig. 1:**
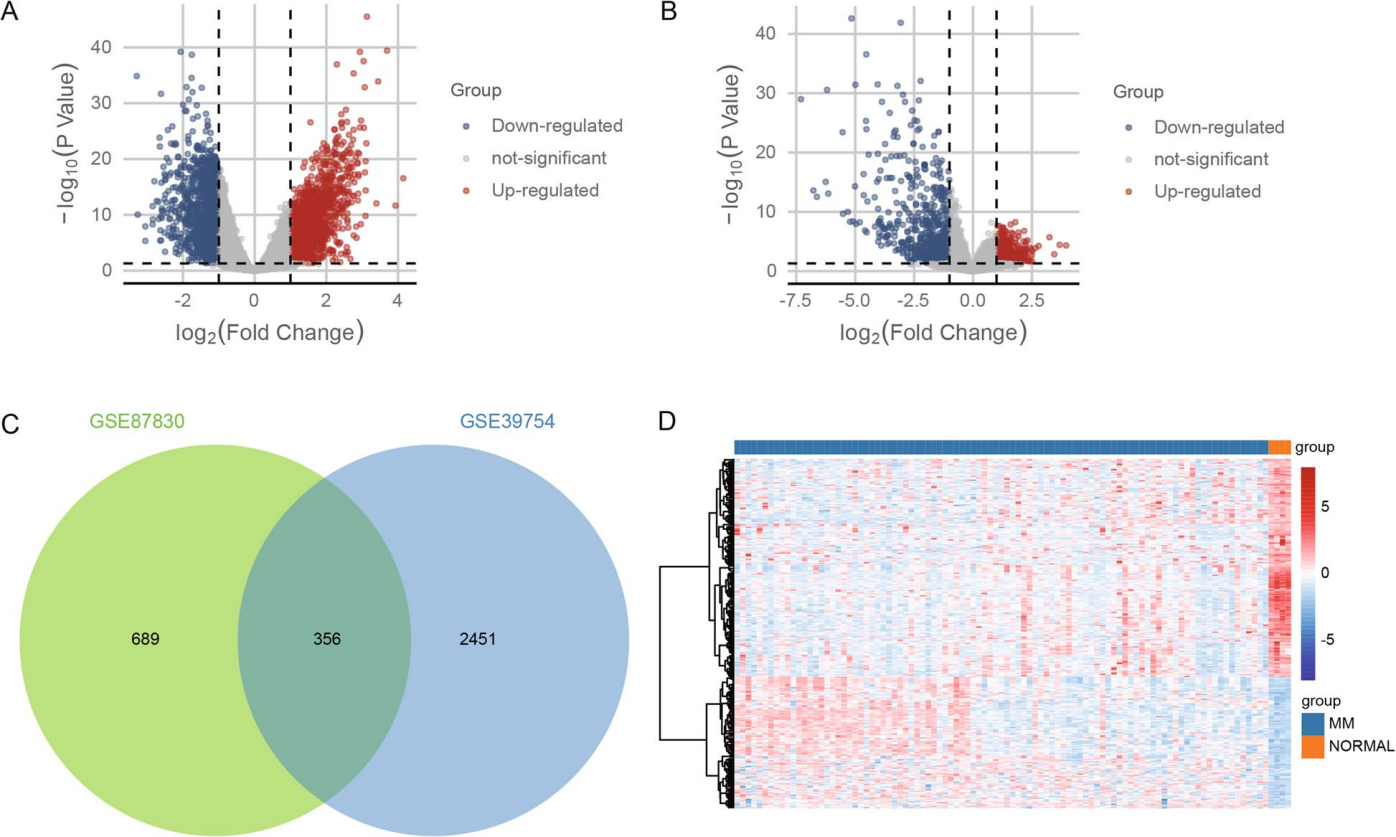
Identification of differentially expressed mRNAs between multiple myeloma and controls. **A** Volcano plot of differentially expressed mRNAs between multiple myeloma and controls in GSE39754. Red indicates upregulation in MM, and blue indicates downregulation. **B** Volcano plot of differentially expressed mRNAs for multiple myeloma and controls in GSE87830. Red indicates upregulation in MM, and blue indicates downregulation. **C** Venn diagram of differentially expressed mRNAs between two DEmRs groups. The intersection includes the common mRNAs. **D** Expression heatmap of common mRNAs in multiple myeloma and control samples of GSE39754

To identify dysregulated mRNAs in MM patients, we performed differential analysis of gene expression profiles of MM and control samples. We obtained a total of 2808 differentially expressed mRNAs in GSE39754 (Fig. 1A). In GSE87830, we obtained a total of 1045 differentially expressed mRNAs (Fig. 1B). Amongst the two sets of DEmRs, we identified 356 common mRNAs with potentially stronger associations with MM (Fig. 1C). The expression of common mRNAs exhibited distinct differences between MM and control samples (Fig. 1D).

### Enrichment of common mRNAs

**Fig. 2:**
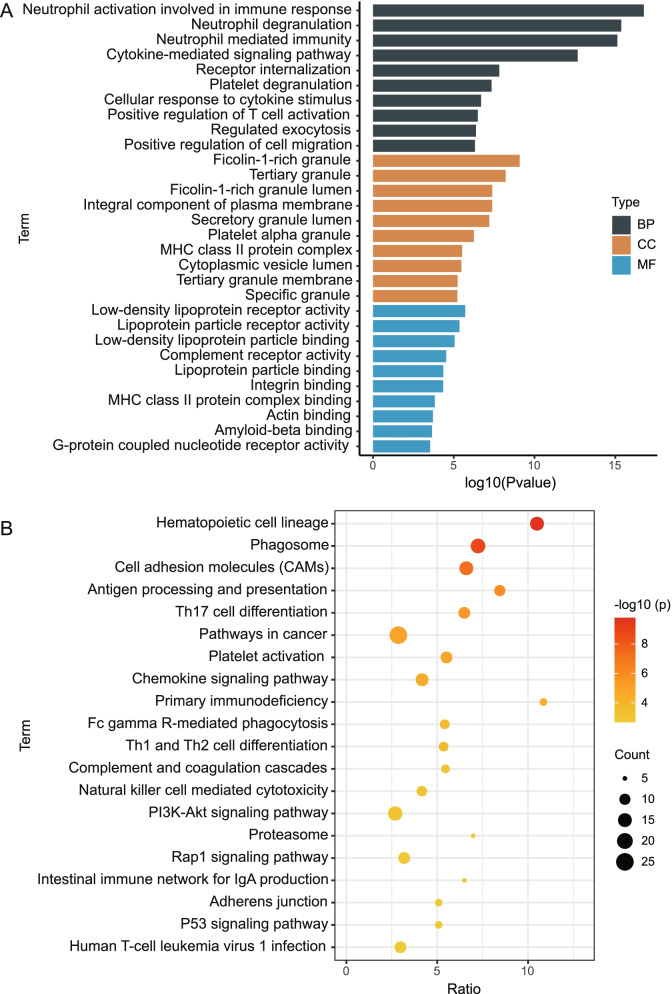
GO functions and KEGG signalling pathways involved in common mRNAs. **A** The primary biological processes, cellular components and molecular functions enriched by common mRNAs. **B** KEGG pathway in which common mRNAs are involved

To identify the biological functions and signalling pathways associated with MM, we performed enrichment analysis on the common mRNAs. GO results indicated that common mRNAs were primarily enriched in biological processes of neutrophil activation involved in the immune response, neutrophil degranulation and neutrophil-mediated immunity (Fig. 2A). In addition, ficolin-1-rich granules, tertiary granules and ficolin-1-rich granule lumens were primarily enriched in cellular components. In molecular function, low-density lipoprotein receptor activity, lipoprotein particle receptor activity and low-density lipoprotein particle binding were enriched. Moreover, KEGG signalling pathway analysis revealed that cell adhesion molecules (CAMs), Th17 cell differentiation and PI3K-Akt signalling pathways were significantly regulated by common genes (Fig. 2B).

### Construction of a miRNA regulatory network

**Fig. 3:**
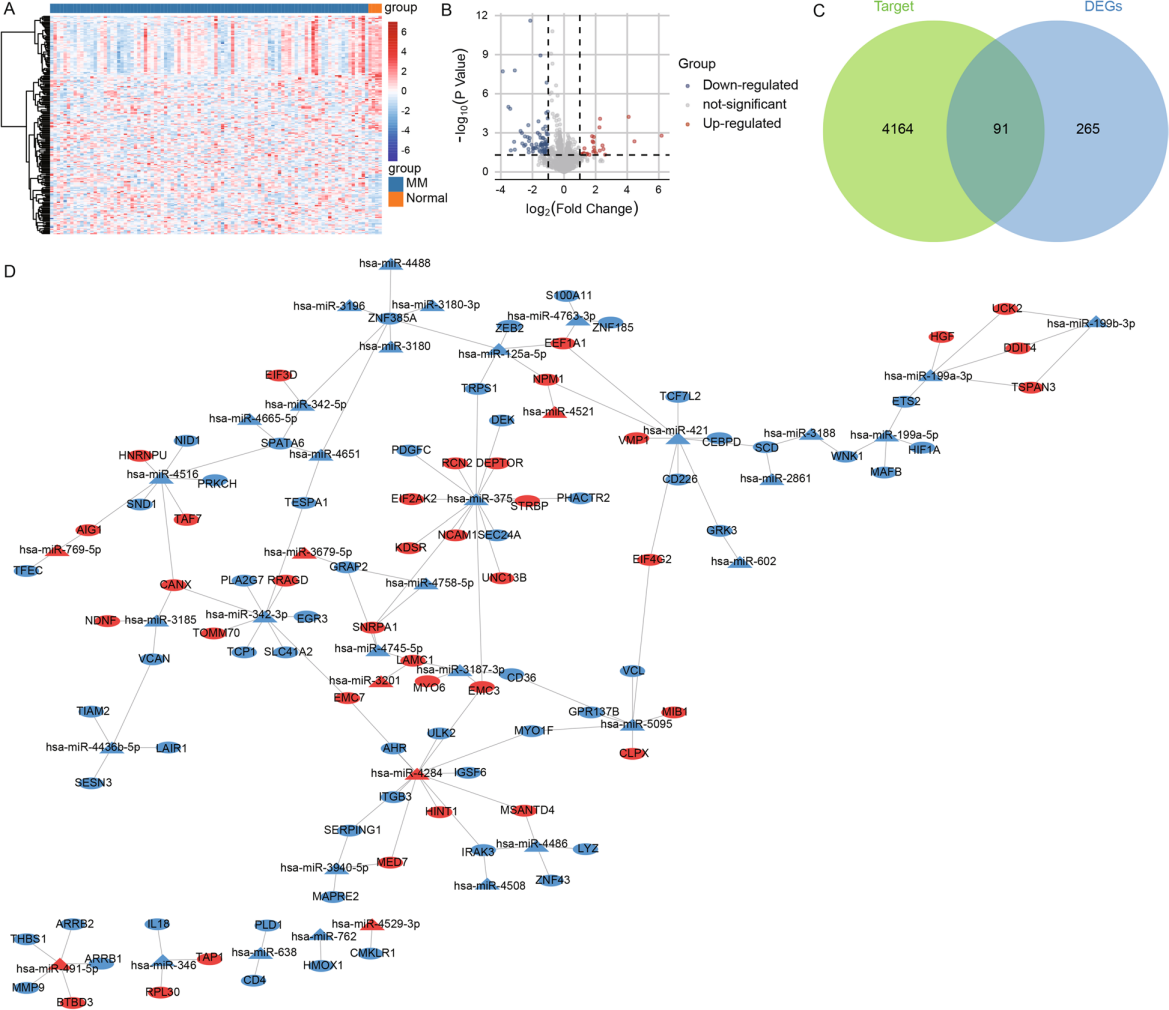
MiRNA-regulated mRNA related to multiple myeloma. **A** Differentially expressed miRNAs between multiple myeloma and controls in GSE87830. **B** Volcano plot of differentially expressed miRNAs for multiple myeloma and controls. Red shows upregulation, and blue shows downregulation. **C** Intersection of target mRNAs predicted using the miRTarget database and common mRNAs. **D** DEmiRs-target common mRNA regulated network. Triangles are miRNAs and ellipses are mRNAs. Red indicates upregulated expression in MM, and blue indicates downregulated expression

In the differential analysis results for GSE87830, we also identified 103 miRNAs that were differentially expressed between MM and controls (Fig. 3A). These included 27 upregulated DEmiRs and 76 downregulated DEmiRs (Fig. 3B). Using the miRTarget database, we predicted 4255 target mRNAs for 103 DEmiRs. By comparison to common mRNAs, we identified 91 differentially expressed target mRNAs (Fig. 3C). Furthermore, we constructed a regulatory network of DEmiRNA target mRNAs (Fig. 3D).

### Identification of key regulatory networks

**Fig. 4:**
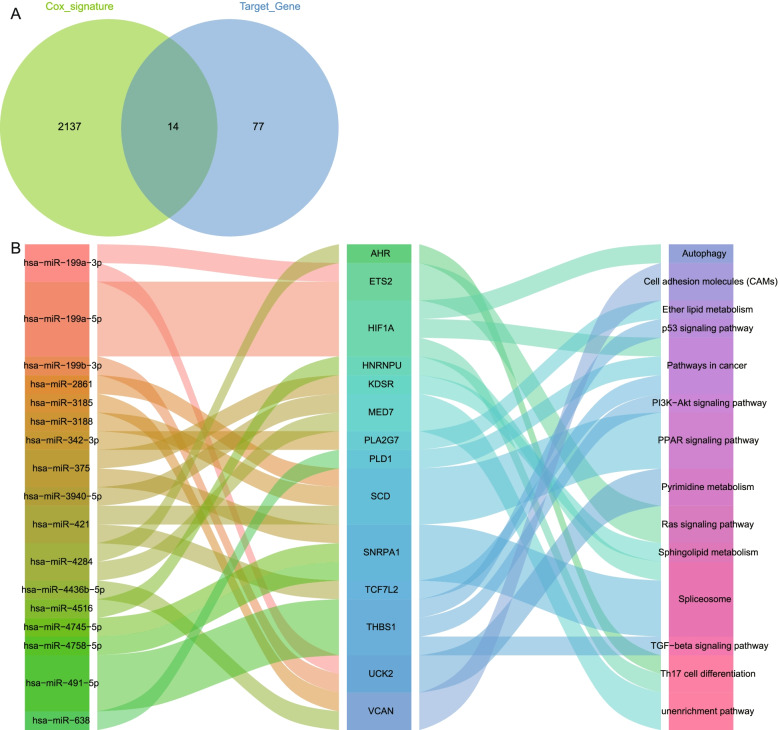
Construction of key miRNA regulatory network. **A** Intersection genes of target mRNAs and mRNAs significantly influencing MM patient prognosis. **B** Comprehensive network of target mRNAs regulated by miRNAs involved in KEGG signalling pathways

Furthermore, using Cox regression analysis, we identified 14 mRNAs amongst the target mRNAs that significantly affected survival in MM patients (Fig. 4A). Amongst them, TCF7L2, PLD1 and PLA2G7 were protective factors, and other mRNAs were risk factors for prognosis of MM patients (Table 1). Finally, we performed a comprehensive analysis of the regulatory network of 14 mRNAs, revealing that hsa−miR−199a−5p regulated HIF1A, and hsa−miR−491−5p regulated THBS1, affecting most KEGG pathways (Fig. 4B). These included significantly enriched Th17 cell differentiation and PI3K−Akt signalling pathways.

**Table 1:** Cox regression analysis of target 14 mRNAs with significant impact on MM patient prognosis

Genes	HR [exp(coef)]	Coef	95% CI lower	95% CI upper	*Z*	*P* value
HNRNPU	1.916105	0.650295	0.460395	0.840195	6.711717	1.92E−11
UCK2	1.431999	0.359071	0.225321	0.492822	5.261785	1.43E−07
AHR	1.150701	0.140371	0.085899	0.194844	5.050696	4.40E−07
HIF1A	1.188999	0.173111	0.10301	0.243212	4.840047	1.30E−06
SNRPA1	1.454355	0.374563	0.194189	0.554936	4.070042	4.70E−05
THBS1	1.111877	0.10605	0.05393	0.158169	3.988022	6.66E−05
MED7	1.438052	0.363289	0.183905	0.542674	3.969322	7.21E−05
TCF7L2	0.792773	−0.23222	−0.34955	−0.11489	−3.87919	1.05E−04
PLD1	0.880664	−0.12708	−0.20639	−0.04776	−3.14021	0.001688
KDSR	1.321984	0.279134	0.101921	0.456347	3.087205	0.00202
VCAN	1.07561	0.072888	0.016381	0.129395	2.528135	0.011467
SCD	1.080565	0.077484	0.016104	0.138865	2.474197	0.013354
PLA2G7	0.943589	−0.05806	−0.10995	−0.00618	−2.1934	0.028278
ETS2	1.064179	0.062204	0.004683	0.119724	2.119549	0.034044

## Discussion

In this study, 356 common mRNAs and 103 DEmiRs were identified, and further examination revealed that these DEmiRs were enriched in the miR-199a-5p/HIF1A and miR-491-5p/THBS1 signalling axes. In addition, some DEmiRs were also enriched in Th17 cell differentiation and PI3K-Akt signalling pathways.

The occurrence of MM changes the expression of many genes, and studying these genes may reveal the mechanism of MM. On the other hand, it also provides new detection standards for early tumour warning, treatment and prognostic evaluation. Screening exogenous agonists and inhibitors for target genes is a major trend in tumour treatment research. Therefore, it is particularly important to screen for key genes that regulate tumorigenesis. In this study, we identified 356 common mRNAs and 103 DEmiRs that may be related to MM. Research on these genes may play a role in the treatment of MM in the future.

The role of miRNAs in cancer progression and their potential application value has received researchers’ attention. Calin et al. reported the association between miRNAs and cancer and identified a dynamic relationship between miRNAs and chromosomes in different types of cancer [18–20]. Due to the relatively stable structure of miRNAs, they have been leveraged as potential tools for cancer diagnosis [21]. Previous studies have also revealed changes in the expression of miRNAs in MM patients and may serve an early warning and play a diagnostic role in the process of MM [15]. For example, miR-34a effectively distinguished between healthy and MM tissues, suggesting that miR-34a can be used as an indicator to diagnose MM [22]. In this study, DEmiRs were enriched in the miR-199a-5p/HIF1A and miR-491-5p/THBS1 signalling axes. In past studies, miR-199a-5p and miR-491-5p were identified as tumour suppressors [23, 24]. However, the effects of miR-199a-5p and miR-491-5p on MM are still unknown. Our results strongly suggested that the miR-199a-5p/HIF1A and miR-491-5p/THBS1 signalling axes may represent key signalling axes in the diagnosis and treatment of MM.

Th17 cells are a subset of helper T cells involved in the process of inflammation and autoimmune disease [25]. In addition, Th17 cells play a regulatory role in tumour progression. Studies have shown that in patients with gastric cancer, Th17 cells are significantly increased in the peripheral blood [26]. Moreover, expression of Th17 cells in peripheral blood changes with tumour progression, which also suggests that Th17 cells can also be used as indicators of tumour prognosis [27]. Moreover, studies have shown that Th17 cells also mediate the occurrence of MM, and the effect of Th17 cells on MM can be eliminated through miR-21 [28]. In this study, DEmiRs were enriched in Th17 cell differentiation, further illustrating the role of Th17 cells in MM.

The PI3K-Akt signalling pathway is an axial pathway that regulates cell proliferation and survival and plays an important role in maintaining vital signs and pathological processes [29, 30]. Similarly, the PI3K-Akt signalling pathway is also involved in tumour progression [31]. Previous studies have revealed that many miRNAs regulate MM through the PI3K-Akt signalling pathway [32–35]. In this study, DEmiRs were significantly enriched in the PI3K-Akt signalling pathway, further supporting the role of miRNAs in MM.

Our study also has some limitations. First, our key results were missing validation in clinical samples. We will utilise a large number of tissue samples for experimental validation in future studies. Thus, the molecular function of key candidate markers remains to be further explored.

## Conclusions

This study preliminarily explored miRNAs as potential diagnostic markers in MM. Further research revealed that DEmiRs were primarily enriched in the miR-199a-5p/HIF1A and miR-491-5p/THBS1 signalling axes. In addition, some DEmiRs were also enriched in Th17 cell differentiation and PI3K-Akt signalling pathways. This study confirmed the role of miRNAs in MM by analysing differences in miRNAs and provides a theoretical basis for future research and application of MM.

## Data Availability

The datasets used and/or analysed during the current study are available from the corresponding author on reasonable request.
